# Role of rumination and hope on negative life events and suicidal ideation under the background of normalization of pandemic prevention and control: A moderated mediation model

**DOI:** 10.3389/fpubh.2022.898580

**Published:** 2023-01-20

**Authors:** Yingying Yao, Zhihong Qiao, Fangbai Dong, Jianchao Ni

**Affiliations:** ^1^Counseling and Education Center, Xiamen University, Xiamen, Fujian, China; ^2^Faculty of Psychology, Beijing Normal University, Beijing, China; ^3^School of Aerospace Engineering, Xiamen University, Xiamen, Fujian, China

**Keywords:** negative life events, rumination, hope, COVID-19, suicidal ideation

## Abstract

**Introduction:**

The study aimed to investigate the impact and mechanism of negative life events on college students' suicidal ideation during the COVID-19 pandemic and the buffering effect of hope under the background of normalization of pandemic.

**Methods:**

A total of 5211 participants took part in this study. Self-reported negative life events, rumination, hope and suicide ideation were measured using a range of questions and scales. Our research demonstrated that the incidence of suicidal ideation among college freshmen in the past week was higher during the COVID-19 pandemic than that before the pandemic. In this study, conditional process model 15 was used to verify the hypothetical model of rumination as a potential mediator and hope as a moderator.

**Results:**

The hypothesized moderated mediation model was verified significant (*β* = -0.047, 95% CI = [-0.061, -0.035]), and hope was found to moderate the direct effect of negative life events on suicidal ideation (*β* = -0.039, t = -2.937, 95% CI = [-0.065, -0.013]) as well as the indirect effect of through the mediator rumination (*β* = -0.134, t = -10.850, 95% CI = [-0.158, -0.110]).

**Discussion:**

We found that rumination partially mediated the effect of negative life events on suicidal ideation, and hope buffered the direct and indirect effect of negative life events on suicidal ideation. The implications of the findings for clinical interventions are discussed, including the importance of hope arousal as a protective factor and rumination as a cognitive mechanism for emotion regulation under the background of normalization of pandemic.

## 1. Introduction

The outbreak of COVID-19, which infected more than 643 million people as of December 2022, had a significant impact across the entire world. As a result, public health departments of governments and other health authorities across the globe have undertaken a wide range of interventions, including nationwide lockdowns, home quarantining, and social distancing, to slow and control the spread of COVID-19. Regarding the impact of COVID-19 and subsequent quarantine on an individual's health, depression and suicidal ideation were seen as the most serious factors, as they have been identified as the factors most strongly associated with suicide (1, 2). College students, in particular, have been considered a vulnerable group who have been found to experience more mental health problems (e.g., depressive symptoms and suicidal ideation) than other adults (3), with neuroticism and stressful experiences as the main factors influencing the increase of suicidal ideation in college students (4). Furthermore, negative life events are considered to be an important indicator of suicidal ideation (5, 6). After the outbreak of the COVID-19 pandemic, college students were faced with stricter campus management, along with the possibility of an increase in negative life events, which further brought severe challenges to college students.

In April 2021, the State Council Information Office of China proposed that the country has entered the normalization stage of epidemic prevention and control (7), with significantly reduced cases of infectious diseases, effective treatment of sporadic infectious diseases, and normal operation of the public health management system. Nevertheless, the impact of COVID-19 continues, and the threat has not been eliminated. Nowadays, the harm of COVID-19 to people's physical health is gradually diminishing, but the impact on people's lives and mental health is ongoing (8, 9). Preventing and eliminating the psychological panic caused by a pandemic is a key issue facing the world today. Under the background of normalized pandemic prevention and control, what is the status of suicidal ideation among college students in the continuous adjustment of the government's prevention and control policy environment? How should suicide crisis intervention be carried out in college and university settings under the background of normalization? In attempting to address these core issues, this study aims to (1) explore the status of suicidal ideation among college students under the background of normalization and (2) discover the influence mechanism of negative life events on suicidal ideation, in order to better advise school counselors and mental health center staffs on early intervention for high-risk populations.

### 1.1. Negative life events, rumination, and suicidal ideation

The stress-diathesis model, proposed by Mann et al. (10), believes that the suicidal risk of an individual is the result of the interaction between an individual's diathesis and the negative life events, including acute mental illness or physical disease, work pressure, adverse interpersonal relationship, severe trauma, and so on. Quite a few suicide theories (10–12) include environmental factors as important antecedent variables of suicidal ideation. Previous studies found that negative life events are important antecedents of depression, despair, suicidal ideation, and even suicidal behavior (13–16). Based on the theories and previous studies, we hypothesized that the combination of physical and mental stress caused by the pandemic (such as worrying about the risk of infection of oneself or the surrounding relatives) and other stressful events may exacerbate suicidal ideation in students.

The integrated motivational-volitional (IMV) model of suicidal behavior (11, 17) proposed that suicide develops in three phases and conducted a detailed theoretical discussion on the relationship between negative life events, rumination, and suicidal ideation. The first phase is the pre-motivational phase, which focuses on distal factors and triggers of suicide risk, including diathesis, environment, and life events. The second phase is the motivational phase, which describes the psychological processes that induce suicidal thoughts and intentions that derive from entrapment triggered by feelings of defeat and humiliation. Self-threatening factors, such as rumination, affect entrapment, as well as the relationship between defeat and entrapment. Rumination is generally considered a maladaptive cognitive process in which an individual repeatedly attempts to analyze problems and painful feelings without taking the necessary action to make positive changes (18, 19).

A growing number of empirical studies also demonstrated that rumination could mediate the impact of negative life events on suicidal ideation (20, 21). Wenzel et al. pointed out that constant immersion in perceptions and influences associated with negative life events might cause individuals to consider suicide, eventually developing a suicidal plan as a solution to their problems (22). Rumination leads to a worsening emotional state, such as more anger and less happiness (23), and has a significant impact on individual mental health, including depressive symptoms (24). The maladaptive nature of rumination is particularly evident in individuals with major depressive episodes (19), and rumination is a risk factor for suicidal ideation (6) and suicide attempts (19, 25). In addition, rumination mediates the impacts of negative life events, childhood trauma, and loneliness on suicidal ideation (6, 26) and even suicidal behaviors (21). Therefore, we hypothesize that negative life events are positively associated with suicidal ideation, and rumination mediates the impact of negative life events on suicidal ideation (Hypothesis 1).

### 1.2. The moderating role of hope on the relationship between negative life events and suicidal ideation

When considering suicidal ideation, some positive psychological structures, such as hope, may be particularly important as protective factors (27). Hope is an individual's mental resilience in the face of future goals (28). Two sub-components of hope comprise the perception of the ability to determine feasible paths to a destination (i.e., pathways thinking) and the perception of being able to use those routes to successfully reach a goal (i.e., agency thinking). Hope, associated with specific goals, can support people to get through persistent negative emotions and, in turn, find meanings out of their sufferings, which has been reported to have protective effects on one's mental health (29). The moderating effect of hope on suicidal ideation has been extensively demonstrated (30–33). Hope moderates the effects of traumatic events on suicidal ideation (30, 32) and alleviates the influence of antecedent factors on suicidal ideation by improving individual coping ability (32). Individuals with a higher level of hope have better-coping strategies (34) and less suicidal ideation (35). Hope theory believes that individuals with high levels of hope will achieve better in various domains, including more positive emotions (28). Hope can also moderate the effect of negative life events on depressive symptoms, one of the most risk elements for suicidal ideation (36). It has been found that improvement of both pathways thinking and agency thinking is beneficial to mental health status (37), and the promotion of hope will be the basic method of adolescent crisis intervention (38). Therefore, based on the hope theory and the buffering hypothesis, it is proposed that hope can moderate the effect of negative life events on suicidal ideation (Hypothesis 2).

### 1.3. The moderating role of hope on the relationship between rumination and suicidal ideation

The IMV model also proposes that motivational variables play moderating roles in the development of depression to suicidal ideation and suicidal intention. Hope, considered one of the motivational factors, can moderate the influence of individual distress on suicidal ideation (33) theoretically. When a goal is blocked, individuals with higher hope are more flexible in seeking and finding alternative paths, which is a powerful mitigating factor for rumination (33, 39). Evoking hope has been found to ameliorate rumination (23). Studies also found that hope moderated the influence of rumination on depressive symptoms in Chinese college students (40). Specifically, rumination significantly predicated depressive symptoms in students with lower hope, but not among students with high hopes (40). Therefore, hope is hypothesized to moderate the effect of rumination on suicide risk (Hypothesis 3).

### 1.4. Present study

The toll of the COVID-19 pandemic on suicidal ideation has been wildly studied, but it is limited in exploring the function of positive psychological structures during the pandemic. There is no specific exploration of suicidal ideation under the context of the normalization of pandemic prevention and control. To bridge the gap, our study examined the association between negative life events and suicidal ideation under the normalization circumstance in Chinese college students and tested the role of rumination as a mediator and hope as a moderator.

**Figure 1:**
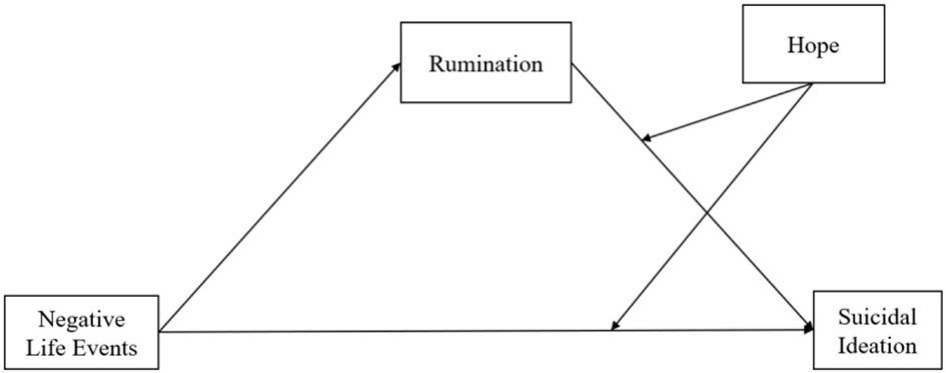
Theoretical hypothesis model-moderated mediation model.

We propose a moderated mediation model as presented in Figure 1 with three hypotheses. First, it is hypothesized that negative life events are positively correlated with suicidal ideation and that rumination plays a mediating role between them. The second hypothesis is that hope moderates the influence of negative life events on suicidal ideation. The third and final hypothesis is that hope buffers the relationship between rumination and suicidal ideation.

## 2. Materials and methods

### 2.1. Participants and sampling

We conducted an online survey of Chinese college students from three universities in Fujian Province during the COVID-19 pandemic using a cross-sectional approach. A mixture of convenience sampling and snowball sampling was used for data collection. A total of 5,632 college students participated in this study, 239 participants did not complete the questionnaire and were excluded, and 182 students were diagnosed with mental disorders and were also excluded, yielding an inclusion rate of 92.52%.

The final sample consisted of 5,211 participants, of whom 55.1% were female. The age ranged from 15 to 38 years (*M*_age_ = 20.74, *SD* = 2.99). There were 2,590 (49.7%) students who majored in natural science, 1,996 (38.3%) students who majored in humanities and social sciences, and 625 (12.0%) students who majored in arts.

Prior to the investigation, participants were notified through the Wechat App about the purpose and procedure of our study. Written consent was obtained online from all participants before accessing the questionnaire.

### 2.2. Measures

#### 2.2.1. The adolescent self-rating life events checklist

Negative life events were measured by ASLEC, containing 27 statements about various life events (e.g., “Relatives and friends suffer from acute or serious illness” and “Being discriminated against”) that may cause psychological distress in adolescents, using a 5-point (0 = no impact, 5 = always impact) Likert scale, and the Chinese version was validated in 1997 (41). A total life stress score was calculated by adding scores on all the items together. The higher the total score, the greater the amount of stress from negative life events. The internal consistency coefficient in the study was 0.947.

#### 2.2.2. Ruminative Responses Scale

Rumination was measured by the Ruminative Responses Scale (42), which consists of 22 items that assess individual responses to depressed mood. Participants reported the frequency of engaging in various ruminative thoughts and behaviors on a 4-point (1 = almost never, 4 = almost always) Likert-type scale (e.g., “I often wonder why I can't get started”). The total score was calculated as the sum of all items with higher scores indicating a greater tendency to ruminate. The internal consistency of RRS in this study was good (Cronbach's α = 0.943).

#### 2.2.3. Adult Dispositional Hope Scale

Hope was assessed using the 12-item Adult Dispositional Hope Scale (43). The Chinese version was validated in 2009 (44). The ADHS is composed of two subscales, namely, pathways thinking and agency thinking. Each subscale consists of four items; for example, the items include “There are always many ways to solve any problem” for pathways thinking and “My experience has prepared me well for my future” for agency thinking. Participants were asked to rate how accurately each item described them on a seven-point Likert-type scale from 1 (absolutely false) to 7 (absolutely true). Higher scores correspond to higher levels of hope. In this sample, ADHS demonstrated good internal consistency (Cronbach's α = 0.872).

#### 2.2.4. Beck scale for suicide ideation

A total of 5 questions were asked to measure the suicidal ideation in the last week from five items rated on a 3-point scale (ranging from 0 to 2): willingness to live, willingness to die, the reasons for survival or death, active suicidal ideation, and the passive suicidal ideation. Higher scores are indicative of stronger suicidal ideation. The internal consistency coefficient of the scale was 0.792.

### 2.3. Data analysis

Correlation analysis was used to check for the bivariate associations between variables. Spearman's correlations were used to analyze non-normal distributions of continuous variables. Descriptive statistics on demographic characteristics and related variables were initially reported. Participants with and without suicidal ideation were compared.

We used the PROCESS Model 15 to test a moderated mediation model with bootstrap confidence intervals for conditional effects. Moreover, a bias-corrected bootstrap procedure was used to generate 95% bias-corrected confidence intervals (CI) from 5,000 samples to test the significance of moderating mediating effects. SPSS 20.0 version was used for the above analyses. The two-tailed significance level was set at *p* < 0.05.

## 3. Results

The items included in all the measures mentioned above were analyzed by exploratory factors, and common method biases were tested using Harman's univariate analysis (45). There were 21 factors with eigenvalues >1. They account for an acceptable percentage (26.34%) of total variation compared to the criterion of 40%, which means no obvious common method bias in our study.

### 3.1. Descriptive statistics and correlations

Table 1 shows the demographic characteristics of the study sample. There was no significant difference in suicidal ideation scores between male and female students (*t* = −0.728, *p* = 0.466), as shown in Table 1. There was a statistically significant difference in the educational level of subjects on suicidal ideation (*t* = 8.58, *p* < 0.001), with the suicidal ideation rate of undergraduates being significantly higher than that of graduates. The differences across majors were statistically significant on suicidal ideation (*F* = 36.569, *p* < 0.001), with the arts students being significantly higher than the other two groupings, and the scores of humanities and social science students were significantly higher than the science and engineering students.

**Table 1:** Demographic characteristics of the study sample.

**Gender**	**Suicidal ideation**	**Education level**	**Suicidal ideation**	**Major**	**Suicidal ideation**
Male (*N* = 2,309)	0.34 ± 0.98	Undergraduate (*N* = 2,571)	0.47 ± 1.18	Natural science (*N* = 2,590)	0.28 ± 0.86
Female (*N* = 2,837)	0.36 ± 1.02	Graduate (*N* = 2,607)	0.23 ± 0.77	Humanities and social sciences (*N* = 1,996)	0.36 ± 1.00
				Art category (*N* = 625)	0.66 ± 1.42
*t*	−0.728	*t*	8.58	*F*	36.569
*p*	0.466	*p*	0.000	*p*	0.000

Table 2 shows the *t*-test results of suicidal ideation in two groups. Students were divided into groups with or without suicidal ideation based on two questions from the BSS-5 (“active suicidal ideation” and “passive suicidal ideation”) as grouping criteria. To be specific, students who said “no” to both two questions were assigned to the group without suicidal ideation; otherwise, they were assigned to another group. The results showed that 501 students had suicidal ideation in the last week, accounting for 9.61% of all the participants. The *t*-test results indicated that the scores of negative life events and rumination in the group with suicidal ideation were significantly higher than those in the group without suicidal ideation (*t* scores were 10.72 and 18.49, respectively, *p* < 0.001), and the scores of hope were significantly lower in the group with suicidal ideation (*t* = −19.21, *p* < 0.001).

**Table 2:** *T*-test of concerned variables between the groups with and without suicidal ideation.

**Variables**	**Suicidal ideation group**	**No suicidal ideation group**	***t***
	**(*****N*** = **501)**	**(*****N*** = **4,710)**	
	***M*** ±***SD***	***M*** ±***SD***	
Negative life events	54.36 ± 20.71	44.08 ± 17.13	10.72^***^
Rumination	2.43 ± 0.62	1.91 ± 0.53	18.49^***^
Hope	2.47 ± 0.47	2.91 ± 0.50	−19.21^***^

*** *p* < 0.001.

Table 3 represents the descriptive statistics and correlation coefficients among all the variables. Suicidal ideation had a significant positive correlation with negative life event scores (*r* = 0.21, *p* < 0.001) and rumination scores (*r* = 0.24, *p* < 0.001) and had a significant negative correlation with hope scores (*r* = −0.26, *p* < 0.001). Negative life events had a positive correlation with rumination (*r* = 0.43, *p* < 0.001).

**Table 3:** Descriptive statistics and correlations between variables.

	***M*** **±*SD***	**Gender**	**Age**	**Negative life events**	**Rumination**	**Hope**	**Suicidal ideation**
Gender	–	1					
Age	20.74 ± 2.99	−0.068^**^	1				
Negative life events	45.08 ± 17.77	0.032^*^	−0.126^**^	1			
Rumination	1.96 ± 0.56	0.059^**^	−0.199^**^	0.43^***^	1		
Hope	2.87 ± 0.52	−0.072^**^	0.180^**^	−0.29^***^	−0.30^***^	1	
Suicidal ideation	0.35 ± 1.01	0.020	−0.124^**^	0.21^***^	0.24^***^	−0.26^***^	1

* *p* < 0.05,

** *p* < 0.01,

*** *p* < 0.001.

We used path analysis to test the direct effect of negative life events on suicidal ideation and the moderating effect of hope on the direct path. After controlling gender and age as covariates, negative life events were found to have a significant effect on suicidal ideation [β = 0.127, *t* = 9.777, 95% CI = (0.101, 0.152)]. Hope negatively predicted suicidal ideation [β = −0.207, *t* = −15.932, 95% CI = (−0.233, −0.182)]. Hope also significantly moderated the effect of negative life events on suicidal ideation [β = −0.097, *t* = −7.771, 95% CI = (−0.121, −0.073)].

### 3.2. Rumination mediated the association between negative life events and suicidal ideation (Hypothesis 1)

To test Hypothesis 1, we used Process Model 4 in PROCESS procedures (46) to detect the mediation model that negative life events influence suicidal ideation through rumination. As shown in Table 4, the path analysis indicated that the direct effect of negative life events on suicidal ideation was significant [β = 0.127, *t* = 9.777, *p* < 0.001, 95% CI = (0.101, 0.152)]. The indirect effect through rumination was significant [β = 0.202, *t* = 14.485, *p* < 0.001, 95% CI = (0.175, 0.229)]. The mediating effect path is shown in Table 4. After adding the mediating variable, the influence coefficient of negative life events on suicidal ideation decreased, and the 95% CI of the mediating effect size [95% CI = (0.057, 0.086)] through rumination did not contain 0, which means rumination partially mediated the association between negative life events and suicidal ideation. The mediating effect accounted for 39.89% of the total effect. The model explained 8.5% of the variation in suicidal ideation. Therefore, Hypothesis 1 was supported.

**Table 4:** Descriptive statistics and correlations between variables.

	**Path**	**Effect**	***SE***	**95% CI**	
				**Lower**	**Upper**
Direct effect	Negative life events-suicidal ideation	0.107	0.014	0.081	0.134
Mediating effect path	Negative life events -rumination -suicidal ideation	0.071	0.007	0.057	0.086
Total effect		0.178	0.013	0.153	0.204

### 3.3. Hope as a moderator (Hypotheses 2 and 3)

To test Hypothesis 2 and 3, we established a moderated mediation model using Model 15 to determine whether the effect of negative life events on suicidal ideation was moderated by hope. The results presented in Table 5 demonstrated that negative life events had a significant positive predictive effect on suicidal ideation [β = 0.077, *t* = 5.781, 95% CI = (0.051, 0.103)]. Rumination had a significant positive predictive effect on suicidal ideation [β = 0.163, *t* =11.890, 95% CI = (0.136, 0.190)]. The interaction between negative life events and hope had a significant predictive effect on suicidal ideation [β = −0.039, *t* = −2.937, 95% CI = (−0.065, −0.013)]. The interaction between rumination and hope had a significant predictive effect on suicidal ideation [β = −0.134, *t* = −10.850, 95% CI = (−0.158, −0.110)]. The results supported Hypothesis 2 and 3. The moderated mediation model was verified significant [β = −0.047, 95% CI = (−0.061, −0.035)], and hope was found to moderate the direct effect of negative life events on suicidal ideation as well as the indirect effect through the mediator rumination. The model explained 14.5% of the variation in suicidal ideation.

**Table 5:** Results of moderated mediation analysis.

	**Suicidal ideation (first step)**			**Rumination (second step)**			**Suicidal ideation (second step)**			**Suicidal ideation (third step)**		
	β	***t***	**95% CI**	β	***t***	**95% CI**	β	***t***	**95% CI**	β	***t***	**95% CI**
Gender	−0.011	−0.436	[−0.058, 0.037]	0.097	4.002	[0.050, 0.145]	−0.009	−0.372	[−0.057, 0.039]	−0.024	−1.011	[−0.070, 0.022]
Age	−0.020	−4.836	[−0.028, −0.012]	−0.055	−13.271	[−0.064, −0.047]	−0.018	−4.339	[−0.027, −0.010]	−0.013	−3.206	[−0.021, −0.005]
Negative life events	0.127	9.777	[0.101, 0.152]	0.351	27.587	[0.326, 0.376]	0.107	7.877	[0.081, 0.134]	0.077	5.781	[0.051, 0.103]
Rumination							0.202	14.485	[0.175, 0.229]	0.163	11.890	[0.136, 0.190]
Hope	−0.207	−15.932	[−0.233, −0.182]							−0.180	−13.940	[−0.206, −0.155]
Negative life events^*^Hope	−0.097	−7.771	[−0.121, −0.073]							−0.039	−2.937	[−0.065, −0.013]
Rumination^*^Hope										−0.134	−10.850	[−0.158, −0.110]
*R* ^2^	0.100			0.169			0.085			0.145		
*F*	114.443			347.885			119.832			123.842		

**Figure 2:**
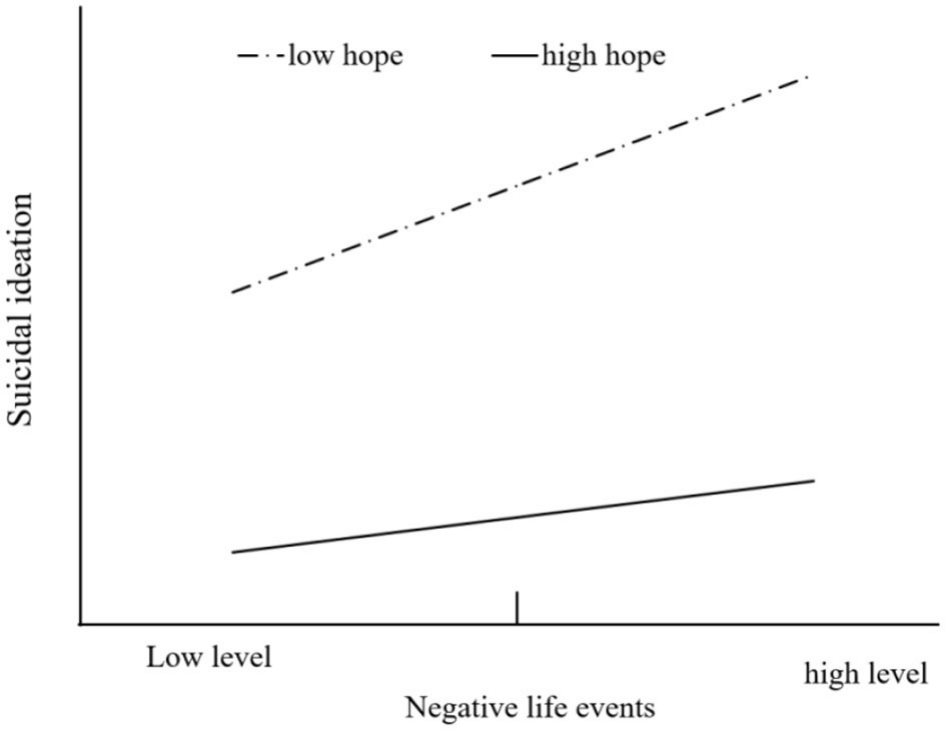
Moderating effect of hope on the relationship between negative life events and suicidal ideation.

The predictive effect of negative life events on suicidal ideation at different levels of hope (1 *SD* above or below the mean) was further examined by simple slopes analysis. The results showed that the positive predictive effect of negative life events on suicidal ideation was significant at a low level (1 *SD* below the mean) of hope [β_simple_ = 0.116, *t* = 6.529, *p* < 0.001, 95% CI = (0.081, 0.150)], and at a high level (1 *SD* above the mean) of trait hope, negative life events had no significant predictive effect on suicidal ideation [β_simple_= 0.038, *t* = 1.907, *p* = 0.057, 95% CI = (−0.001, 0.077)], which indicates that a higher sense of hope could mitigate the effects of negative life events on suicidal ideation. A simple effect slope diagram is shown in Figure 2.

**Figure 3:**
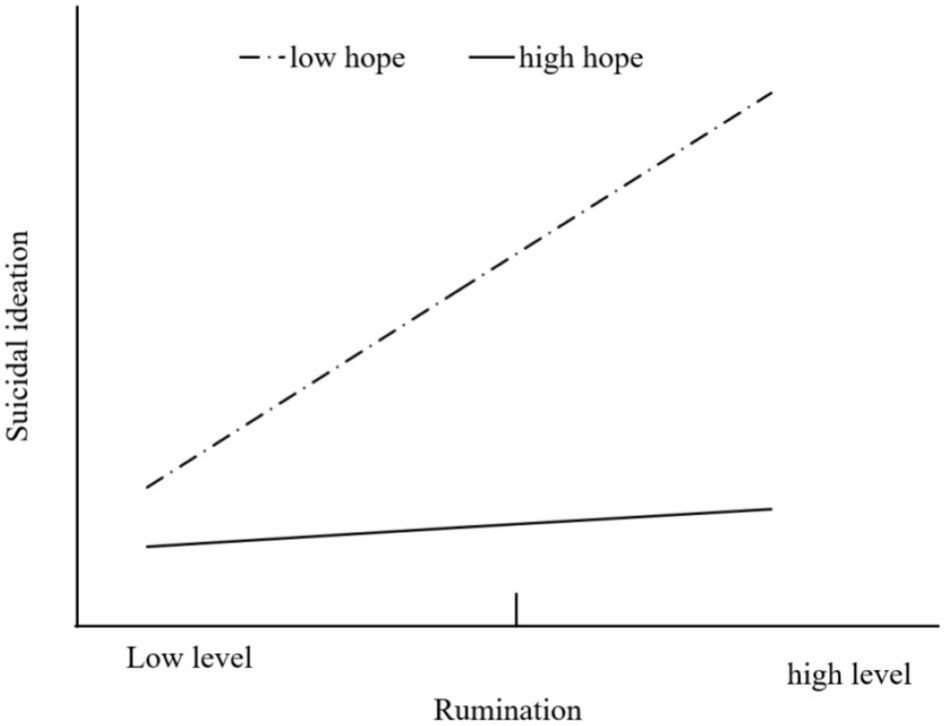
Moderating effect of hope on the relationship between rumination and suicidal ideation.

According to the findings of simple slopes analysis, we further examined the predictive effect of rumination on suicidal ideation at different levels of hope (1 *SD* above and below the mean). The results showed that rumination had a significant positive predictive effect on suicidal ideation at low levels of hope [β_simple_ = 0.295, *t* = 16.286, *p* < 0.001, 95% CI = (0.260, 0.331)], but at high levels of hope, rumination had no significant predictive effect on suicidal ideation [β_simple_ = 0.029, *t* = 1.523, *p* = 0.128, 95% CI = (−0.008, 0.065)]. The additional results of the slopes analysis (Figure 3) indicated that the slope of the indirect effect was greater for a lower level of hope than for a higher level of hope, which means that hope could mitigate the effects of rumination on suicidal ideation.

In addition, hope was found to moderate the indirect effect of negative life events on the prediction of suicidal ideation through rumination, and the moderated mediating effect size was −0.047 with 95% CI (−0.061, −0.034). At a low level of hope (1 *SD* below the mean), the indirect effect of negative life events on suicidal ideation through rumination was significant [β = 0.104, 95% CI = (0.080, 0.128)]. At a high level of hope (1 *SD* above the mean), the indirect effect of negative life events on suicidal ideation through rumination was not significant [β = 0.010, 95% CI = (−0.001, 0.021)].

## 4. Discussion

Our study focuses on the suicide ideation of college students under the context of normalization of pandemic prevention and control in China. It was a meaningful perspective and expansion on distinguishing the potential detailed difference between suicidal ideation during the outbreaking period of the pandemic and suicidal ideation during the normalization period of it. It verified the mediating role of rumination, which is consistent with the previous study during the outbreaking period of COVID-19 (6). Second, our study also explored the moderating effect of hope on negative life events and rumination. It enriches the research of hope theory under the background of the COVID-19 pandemic. It complemented the previous study in suicidology that did not pay enough attention to the positive psychological structure of individuals, especially during the pandemic, and it also promoted the exploration and enlightenment of the suicide prevention mechanism for college students from the perspective of hope.

### 4.1. The suicidal ideation of college students under the context of normalization of pandemic prevention and control

This study focuses on the suicidal ideation of college students under the context of normalization of pandemic prevention and control. In our studies, the prevalence of suicidal ideation among college students in the past week was 9.61%, which was much higher than the data reported on suicidal ideation (2.60–6.18%) (47). Another study showed that the prevalence of suicidal ideation rose from the early phase (7.6%) to the later phase (10.0%) during the COVID-19 pandemic. A cohort study of employees from Japan and a longitudinal study of adults in the United Kingdom also reported an increase in the incidence of suicidal ideation during the COVID-19 pandemic (48, 49). Perhaps due to long-time isolation and restrictions, college students feel confused and fearful about their future career or academic development, which causes a lot of mental health problems (50). In conclusion, these findings suggest that suicidal ideation has become an even more important and urgent public health problem due to the COVID-19 pandemic (49, 51). Therefore, there is a need to improve suicide prevention strategies during this period of time. In the mitigation period of the pandemic, colleges and universities have exercised more stringent control over collective activities, thereby reducing collaborative opportunities, such as team building, class integration, and community activities. Limited interpersonal communication and occasional outbreaks may contribute to adjustment difficulties and emotional distress in college students. Given the characteristics of the pandemic remission period, colleges and universities need to make further adjustments in the provision of mental health services, such as popularizing mental health knowledge through the media, increasing awareness of and access to psychological help hotlines, and launching online psychological counseling services to meet the psychological needs of students in a timely fashion.

### 4.2. Buffering effect of hope on risk factors and suicidal ideation

This study explored the influence mechanism of negative life events on suicidal ideation in college students, to seek a feasible direction for crisis intervention efforts. Based on the IMV model of suicidal behavior, we used the moderated mediation model to verify the influence of negative life events on suicidal ideation. It was found that rumination partially mediates the effect of negative life events on suicidal ideation, and the mediating effect is moderated by hope, which provides some ideas for reducing suicidal ideation among college students.

First, rumination was verified to mediate the relationship between negative life events and suicidal ideation, and the mediating effect accounted for 39.89% of the total effect, which reveals the importance of cognitive interventions directed at individuals' cognitions. In the context of the pandemic mitigation period, the challenges of adapting to and integrating into the new environment have become more severe. Faced with negative life events, college students often engage in rumination and focus on negative emotions and thoughts about adverse consequences, which tends to increase suicidal thoughts. Therefore, it is vital to intervene with college students' suicidal ideation from the perspective of their cognitions. For example, helping students gain access to mental health information and resources could reduce their adjustment difficulties, thereby reducing their repeated immersion in negative thinking. In addition, guiding students toward a more rational understanding of the occasional outbreak rather than being trapped in negative and pessimistic emotions, communicating with them timely, and correcting possible cognitive biases and fixations will help to decrease students' suicidal ideation (52). In addition, it is strategically important to understand the practical problems of students and reduce their rumination by exploring more adaptive cognitive strategies.

Second, we found that hope buffered the effect of negative life events on suicidal ideation. Individuals with higher levels of hope are expected to adopt more positive and practical strategies to achieve the desired goals (28). They are able to take a broader perspective and tend to embrace positive reactions to negative life events. Previous studies have demonstrated that activating hope can significantly reduce depressive symptoms (53), which is an important prevention in suicidal ideation. College students may experience excessive stress due to academic and employment preparation, financial difficulties, and strained interpersonal relationships (54). Keeping hope under stressful circumstances may promote the insight that there are difficulties to overcome or targets to achieve (agency), rather than only seeing barriers. In this manner, motivation to deal with stressors may increase, along with the awareness of pathways and the self-confidence and pathways to achieve goals (43). This, in turn, may reduce the painful effects caused by the experience of negative life events and ultimately reduce symptoms of depression and suicidal ideation. Research shows that individuals with high hope levels have higher self-esteem, higher self-efficacy, and better behavior than individuals with low hope levels (31). Hope is a kind of positive mental quality that can offer flexible resources for individuals to effectively respond to risk events, thereby reducing the adverse effects of these events.

It was also found that hope moderated the indirect effect of negative life events on suicidal ideation through the mediating role of rumination. Some scholars have proposed that there are two dimensions of rumination, namely, obsessive thinking and introspection (25). Obsessive thinking is considered maladaptive for the clue that it can lead to the onset of depression, prolong the duration of depression, and worsen the degree of depression. Hope provides a powerful psychological capital that may change the nature of rumination from maladaptive to adaptive. Specifically, rumination in individuals with high levels of hope is more likely to have a positive adaptive function. Even when there is a non-adaptive obsessive thought (such as “Why can't I do things better”), he actively makes full use of this thinking to generate greater motivation and find better ways to solve problems. Conversely, rumination may have negative and undesirable functions in individuals with low hope. For example, people with low hopes adopt a perturbative approach (e.g., “I was distracted by certain thoughts”), and they will interpret the situation in a negative way. For example, they may think they are incapable of solving the problem and therefore eventually give up. Thus, hope plays an important role in determining whether rumination is adaptive or maladaptive.

### 4.3. Practical implications

In this study, the strong inverse association between hope and suicidal ideation and its buffering effect predicted the potential value of hope as a target for treatment and intervention. For example, group therapy (55) focused on hope arousal (23) was found to be effective in promoting the level of hope and cultivating students' positive psychological state. As face-to-face activities may be restricted to varying degrees during the pandemic mitigation period, university staff are encouraged to use multimedia platforms to promote the information students need. Targeted online group interventions are encouraged, especially focusing on the cognitive level and specifically targeting rumination, in order to reduce rumination levels and enhance the hope of coping with the difficulties encountered in the new adjustment period. In addition, interpersonal relationships are also an important source of hope. Colleges and universities are encouraged to build a small but intimate social support system with dormitory parks and dormitories as a fulcrum. This can enhance students' sense of belonging and connection. The results provide a positive psychological perspective for the practice of crisis intervention in colleges and universities and additionally provide empirical support for the need for further research on the positive psychological effects of inspiring students to better with suicidal ideation.

After accounting for the effect of rumination, negative life events can still directly influence suicidal ideation. Cognitive change alone is not enough to prevent suicidal ideation in college students. An important direction for the primary prevention of college students' crises prevention is to guide students to solve realistic problems, make better use of resources, and become familiar with the new environment as soon as possible. During the remission period of the pandemic, colleges and universities should assess the difficulties and challenges faced by college students in advance, sort out the ways of obtaining various resources as fully as possible, provide varied forms of help in the process of students' adaptation, and adjust the enrolment education program to minimize the practical difficulties and realistic pressure faced by students.

### 4.4. Limitations

Several limitations of our study should be noted. First, cross-sectional studies do not allow us to establish temporal priorities among target variables or to make causal inferences. Therefore, longitudinal studies should further examine the effect of rumination on depression or suicidal ideation in the future. Second, all participants were from Fujian Province, China. However, in terms of outbreak management, it is understood that responses may differ across provinces due to geographical management. The implications of the current conclusions for college students in other provinces in China, and even other countries, need further research. Third, data on incoming college students in the same region prior to the COVID-19 outbreak were not collected, so changes in the mental health status of college students referred to previous studies for baseline comparisons. Follow-up studies would be best used to track changes in suicidal ideation among college students nationwide to better confirm the impact of COVID-19 on mental health status.

## 5. Conclusion

Our results found that rumination partially mediated the effect of negative life events on suicidal ideation and that hope buffered the direct and indirect effects of negative life events on suicidal ideation, which means that the negative effects of negative life events and rumination on suicidal ideation may be reduced if individuals have higher levels of hope. Based on the results, this study provides practical suggestions for guiding college students' attempts to cope with negative life events, intervening for reflection, cultivating and enhancing students' hope, and providing theoretical support for an approach to the primary prevention of secondary and tertiary interventions with college students who may or are experiencing suicidal ideation during the remission period of the pandemic.

## Data availability statement

The original contributions presented in the study are included in the article/supplementary material, further inquiries can be directed to the corresponding author.

## Author contributions

YY designed the study, gathered the data, performed the analysis, and interpreted the data. YY and FD drafted the manuscript. YY, ZQ, FD, and JN revised the manuscript. All authors have read and agreed to the published version of the manuscript.
